# Evolutionary Trends in Integrated Care in 2000–2020 in China: A Scientometric Review Using CiteSpace

**DOI:** 10.5334/ijic.6006

**Published:** 2022-09-23

**Authors:** Xiatong Ke, Liang Zhang, Wenxi Tang

**Affiliations:** 1 Research Department III, Shenzhen Health Development Research and Data Management Center, Shenzhen, Guangdong, 518000, PR China; 2 School of Political Science and Public Administration, Wuhan University, Wuhan, Hubei 430030, PR China; 3 Research Center for Rural Health Services, Hubei Province Key Research Institute of Humanities and Social Sciences, Wuhan, Hubei 430030, PR China; 4 School of International Pharmaceutical Business, China Pharmaceutical University, Nanjing, Jiangsu 211198, PR China; 5 Center for Pharmacoeconomics and Outcomes Research of China Pharmaceutical University, Nanjing, Jiangsu 211198, PR China

**Keywords:** integrated care, visual analysis, CiteSpace, scientometrics

## Abstract

**Introduction:**

Owing to an increasing demand for a continuous and coordinated health service, integrated care is being promoted worldwide. Chinese research on integrated care has rapidly increased over the last 20 years. However, popular topics, paths and trends of integrated care research in China have not been systematically summarised. The study aimed to examine the evolution of integrated care research in China and predict future research trends.

**Methods:**

We searched for integrated care research in China published 2000–2020 in Chinese (China National Knowledge Infrastructure) and English (Web of Science). Research articles that met the inclusion criteria were selected. CiteSpace 5.7.R3 was used to perform keyword clustering, timeline view and burst detection analyses.

**Results:**

We included 786 Chinese articles and 124 English articles. Chinese articles formed 10 clusters with 1814 keywords. English articles formed 5 clusters with 487 keywords. From 2000 to 2020, integrated care research in China comprised three stages: (1) In the start-up stage (2000–2007), keywords mainly focus on medical resource integration and two-way referral; (2) In the emergence stage (2008–2015), keywords primarily include integrated model, benefits of integration, paths to integration and incentive mechanisms; (3) In the maturation stage (2016–2020), the main keywords are patient preferences, shared management mechanisms, symbiosis theory, value-based care, payment methods and people-oriented care.

**Discussion:**

With increasing health care system reform, popular integrated care research topics in the next stage will likely focus on people-oriented integrated care, service value and payment method reform. Academic research on integrated care in China will help to shape and lead policymaking.

**Conclusions:**

Integrated care research in China has gone through three stages over the last two decades. In the future, integrated care theory in China will be informed by concepts from other fields, such as value co-creation in public management, to address the current problem of lack of synthesis in integrated care in China.

## Introduction

In recent years, in the context of increasing health threats in China and health care system challenges, the Chinese government and researchers have paid more attention to the concept of integrated care. Since 2015, the Chinese government has successively issued documents at the central government level, such as the ‘Guiding Opinions on Promoting the Construction and Development of Medical Consortia (issued by the General Office of the State Council [2017] No. 32)’ and the ‘Notice on Issuing the Measures for the Administration of Medical Consortia (for Trial Implementation) (issued by the General Office of the National Health Commission (2020) No. 13)’. These documents outline plans to promote the development of medical consortia, medical communities, specialist alliances and the integration of medical care and prevention. The main measures include establishing urban medical groups and county-level medical communities using a grid-based layout, facilitating the construction of specialist alliances for major diseases and medical resources in short supply, and providing personalised family physician contracted services according to residents’ needs. The interest of Chinese researchers in integrated care precedes that of governmental interest. In the 1980s, Chinese researchers began to study the embryonic form of integrated care – the medical consortia. However, the pace of theoretical research and practice has been slower since then, focusing on vertical organizational integration and resource integration of health care institutions at all levels within a region for integrated care. In terms of content, the focus has been on the service provision of health care institutions at all levels in the region, especially the sharing of technology and human resources; in terms of model, the focus has been on the vertical integration between different levels of health care institutions; in terms of level, most of the research and practice on integrated care in China has stayed in the macro level, and no research on the integration of single-patient health care services or the integration of services for specific populations has been found. In recent years, Chinese scholars have begun to focus on the effectiveness and efficiency of integrated care [1]. For example, the KE (2021) study explored the costs and benefits of an integrated care model using multidisciplinary team (MDT), multi-institutional pathways (MIP), and system global budget and performance-based payment (SGB-P4P) for chronic disease management in China [2].

As in many other countries, research on integrated care in China is growing rapidly [3,4,5,6], but a unified understanding of integrated care is lacking [7]. Integrated care is a broad concept that generally refers to the integration of various health care and the coordination of medical institutions at different levels to provide lifelong coherent services for the people, such as health promotion, disease prevention, diagnosis and treatment, nursing rehabilitation, and hospice care. Some researchers have also given their understanding of the concept of integrated care, either focusing on one aspect of the above definition or extending the concept further to the integration of services in the health field with other social fields. For example, Gröne [8], Kodner [9], and others focused on the financing, organization, management, and service delivery processes of the subsystems within the health system. Enthoven A C [10] further refined the process of organizing and delivering services by showing that this needs to be achieved through collaboration, the interaction between different levels of health services/medical staff, in the form of setting up branches, alliances, or contracts. Lewis [11] emphasized that the primary goal of integrated care is to improve service quality and patient satisfaction, and to increase the cost-effectiveness of services; Leutz [12] emphasized that integrated care required collaboration not only with subsystems within the health system but also with other social systems (e.g., education, housing, etc.) from a “big health” perspective.

No studies have systematically summarised the popular topics, paths and trends of research on integrated care in China. Therefore, information about the history, current status and future trends of integrated care research in China remains incomplete. Therefore, this study was conducted to systematically clarify the disciplinary base, main research paths and future research trends in the field of integrated care in China from 2000 to 2020. We used scientometric methods, including keyword co-occurrence analysis, timeline view analysis and burst detection analysis, with CiteSpace 5.7.R3 software.

## Methods and Data

### Introduction to CiteSpace Software

CiteSpace (Citation Space) is a diversified, time-sharing and dynamic citation visual analysis software. It focuses on the analysis of potential knowledge in scientific literature, and has gradually developed against the background of scientometrics and data and information visualisation [13]. The CiteSpace software is based on Thomas Kuhn’s theory and model of scientific development, Price’s scientific frontier theory and structural holes, Kleinberg’s burst detection algorithm and the optimal information foraging theory of scientific communication [14]. CiteSpace uses multiple functions, including the discovery of disciplinary turning points, the detection of research frontiers and the visualisation of transmission routes for popular topics in disciplinary research.

### Analysis Indices

Using CiteSpace 5.7.R3, an intuitive knowledge map analysis was conducted for keywords in research on integrated care in China. The scientometric analysis methods used included cluster analysis of keyword co-occurrence, timeline view analysis and burst detection analysis.

Keyword co-occurrence cluster analysis counts the number of times a specific set of keywords occurs in the same set of articles. Based on the frequency of keyword occurrence in the articles, a co-word matrix is constructed and cluster analysis is performed to generate a co-occurrence network. This identifies popular topics in a specific research field [15].

The timeline view focuses on delineating the relationship between clusters and the historical span of articles in a specific cluster. Keywords in the same cluster are placed on the same horizontal line in chronological order. Therefore, the keywords of each cluster appear on a timeline showing the historical progress of the cluster. The time span of different clusters, together with the rise, peak and decline of research in a particular cluster, is obtained to further explore the temporal characteristics of the research field reflected by clustering [15].

Burst detection analysis mainly uses the algorithm proposed by Kleinberg (2002) for detection [16]. In CiteSpace, a high number of burst nodes in a cluster indicates an active research field or an emerging research trend. In addition, betweenness centrality is used to measure the importance of keywords in each cluster. The method for calculating the importance of keyword nodes was proposed by Freeman in 1977, and the calculation of betweenness centrality is as follows [17]:


{\rm BC}_{i} = \sum\limits_{s \ne i \ne t} {\frac{{n_{st}^i}}{{{g_{st}}}}}

where *g_st_* is the number of shortest paths from node *s* to node *t*, and n_{st}^i is the number of shortest paths that pass node *i* among *g_st_* shortest paths from node *s* to node *t*. From an information transmission perspective, higher betweenness centrality is associated with greater node importance and a greater effect on network transmission after removal of these nodes.

However, the analysis method of this article has limitations. The co-occurrence analysis in this paper is based on keyword co-occurrence rather than references, mainly because CiteSpace only supports keyword analysis for the China National Knowledge Infrastructure (CNKI) database at present. Since references are not included in the data exported from the CNKI, the current study could not perform reference co-occurrence analysis on Chinese language literature. By studying the CiteSpace software operation guide, we summarize that to complete the co-occurrence analysis of CNKI, three problems need to be solved: how to obtain references, how to convert the data format of references, and how to write references into the data file for co-occurrence analysis. The idea of solving these problems is to obtain references manually first, and then combine python programming to write data literature on references, and finally achieve co-occurrence analysis of Chinese language literature in the CNKI database. However, the number of references is huge, and manual acquisition requires a lot of labour and time, which is difficult to realize. Although co-occurrence analysis of references cannot be used, keywords co-occurrence analysis has been able to meet the aims of our research. Keywords are the core summary of a paper, and co-occurrence analysis of keywords in a paper can determine the hot topics of research in each subject represented in the literature set. Secondly, by counting the frequency of keywords appearing in the same paper between a group of papers, a co-occurrence network consisting of these word pair associations can be formed.

### Data Collection

The data sources for this review were China National Knowledge Infrastructure (CNKI; https://www.cnki.net/) and Web of Science (http://www.isiknowledge.com/). The two databases were used to search the Chinese and English language literature, respectively, for previous disciplinary knowledge bases, current popular research topics and future research trends in the field of integrated care in China.

#### Chinese Language Literature

The literature search of the CNKI database was performed using the following method: full text = ‘integrated health care’ (in Chinese: ‘整合卫生服务’); discipline = research on principles, policies, laws and regulations of medical and health care; language = Chinese; literature type = research article; time span = 2000–2020. The exclusion criteria for the literature were (1) documents irrelevant to the research subject, such as conference notices, news reports and advertisements; (2) republished documents; and (3) documents irrelevant to the subject of integrated care, such as the integration of urban and rural medical insurance systems, public hospitals, information construction, hospital management, integrated medicine and health and family planning.

#### English Language Literature

The following search strings were used to search the Web of Science Core Collection: #1: ALL = (‘Integrat* in China’ OR ‘Integrat* care in China’ OR ‘Integrat* service in China’ OR ‘Integrat* health care in China’ OR ‘Integrat* delivery in China’ OR ‘Integrat* delivery system in China’), time span = 2000–2020, with 39 records retrieved; #2: TS = (‘Integrat*’ OR ‘Integrat* care’ OR ‘Integrat* service’ OR ‘Integrat* health care’ OR ‘Integrat* delivery’ OR ‘Integrat* delivery system’) AND AD = (‘China’) AND WC = (‘Health care science services’ OR ‘Health policy services’), time span = 2000–2020, with 87 records retrieved; and #3: #2 OR #1, with 124 records retrieved. Subsequently, all literature data were imported into CiteSpace 5.7.R3 software for analysis.

### Data Analysis

#### Data analysis of Chinese language literature

All Chinese articles selected from the CNKI database according to the search strings and the inclusion/exclusion criteria were imported into CiteSpace 5.7.R3, and the data format was converted in accordance with the software requirements. The parameters were set in CiteSpace 5.7.R3 as follows: (1) Time Slicing region: set the time range to 2000–2020, with each year as a time slice; (2) Term Source region: select Title, Abstract, Author Keywords (DE), and Keywords Plus (ID); (3) Node Types region: select Keywords; (4) Links region: select the default parameters; (5) Selection Criteria region: after several rounds of parameter adjustments, set the threshold selection to Top 60 when performing keyword co-occurrence analysis to achieve the best clustering effect; this indicates that the top 60 data pieces of each time slice were extracted to generate the final network; (6) Pruning region: check Pathfinder and Pruning sliced networks (Pathfinder helps to simplify the network and highlight its important structural features) [18].

#### Data analysis of English language literature

When using CiteSpace to analyse the English language data retrieved from the Web of Science database, the parameter settings for Time Slicing, Term Source, Node Types, Links, Pruning and other regions were consistent with those described above, except that the Selection Criteria region settings were different. To obtain a more scientific and logical map, the threshold parameter was determined after multiple adjustments. When performing keyword co-occurrence analysis, the threshold was set to g-index k = 25; the g-index takes into account the citation frequency of articles. Larger g-index values indicate a greater influence of researchers and higher academic achievement.

## Results

A total of 2532 Chinese language records were retrieved from the CNKI database based on the search strings, and 786 Chinese articles were finally included according to the inclusion criteria. In addition, 124 English language articles were retrieved from the Web of Science database. After analysis by CiteSpace 5.7.R3, the Chinese articles formed 10 clusters with 1814 keywords; the English articles formed five clusters with 487 keywords.

### Visual analytics of the Chinese language literature

#### Keyword clustering and timeline view analysis

**Figure 1:**
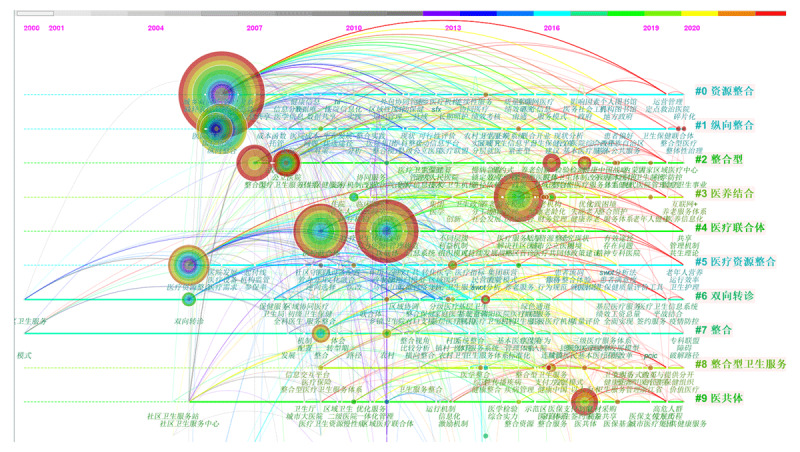
Timeline view of integrated care research in China.

The timeline view obtained after keyword clustering is shown in Figure 1 and the content of all 10 clusters is shown in Table 1. Based on the timeline view, the network modularity Q = 0.6234 > 0.3, indicating that the clustering was significant. The mean silhouette S = 0.8361 > 0.7, indicating that the cluster members had a relatively high consistency and that the result was convincing. Moreover, the keyword nodes N = 1222, connection number E = 3616 and network density = 0.0048. The largest CC represents the information of members in the largest subnetwork; 1126 indicates that the largest subnetwork of the network had 1126 members, accounting for 92% of the total nodes. Overall, the keywords with high co-occurrence frequencies were ‘resource integration’ (108 times), ‘medical consortium’ (97 times), ‘hierarchical diagnosis and treatment’ (93 times), ‘combination of medical and elderly care’ (75 times), ‘longitudinal integration’ (63 times) and ‘integrated care’ (30 times).

**Table 1:** Summary of thematic concentrations in the Chinese language literature.


CLUSTER	SIZE	SILHOUETTE	AVERAGE YEAR	KEYWORDS BURSTNESS

#0 Resource integration (资源整合)	105	0.847	2013	2006–2010: Urban–rural two-way referral (城乡双向转诊) and health information (健康信息). 2010–2015: Coordinated management (协调管理), continuous service (联续性服务). 2016–2020: Resource Integration Practice (资源整合实践), rural medical insurance system (农村医疗保障体系), doctor–patient relationship (医患关系);

#1 Longitudinal integration (纵向整合)	88	0.889	2013	2005–2014: Hospital management (医院管理), medical resources (医疗资源), longitudinal integration (纵向整合), index system (指标体系); 2015–2019: Medical alliance (医疗联盟), two-way referral (双向转诊), rural health care (农村卫生服务), hospital group (医院集团); 2019–2020: Fragmentation (碎片化), patient preference (患者偏好), health care consortium (卫生保健联合体), general ward (综合病房), payment system (付费制度), counterpart support (对口帮扶), multidisciplinary team (多学科团队);

#2 Integrated type (整合型)	83	0.868	2014	2007–2010: Public hospital reform (公立医院改革), integrated health care service (整合型医疗卫生服务); 2010–2016: County-level medical community (县域医共体), medical consortium (医疗联合体), medical group (医疗集体); 2016–2020: Senior care innovation (养老创新), compact type (紧密型), and specialist alliance (专科联盟), etc.;

#3 Combination of medical and elderly care (医养结合)	81	0.844	2016	2013–2016: Combination of medical and elderly care (医养结合), innovative elderly care model (创新养老模式), healthy aging (健康老龄化), healthy elderly care (健康养老), long-term elderly care (老年长期照护), and community home (社区居家), etc.; 2016–2019: Optimisation of practical difficulties (优化实践困境), integrated care of disabled elderly (失能老人整合照护), and quality of life (生命质量), etc.; 2020: Informatisation of medical and elderly care (医养信息化), Internet+ (互联网+), and function integration (功能整合), etc.;

#4 Medical consortium (医疗联合体)	80	0.814	2015	2008–2015: Integration and optimisation (整合优化), hierarchical diagnosis and treatment (分级诊疗), organisational model (组织模式), and benefit mechanism (利益机制); 2015–2020: Hospital reorganisation (医院重组), chronic disease prevention and treatment (慢病防治), symbiosis theory (共生理论), corporate governance structure (法人治理结构), and payment system reform (支付制度改革), etc.;

#5 Integration of medical resources (医疗资源整合)	78	0.869	2013	2007–2020: Medical resource integration (医疗资源整合), performance appraisal (绩效考核), continuous service (连续性服务), single disease (单病种), system construction (制度建设), data sharing (数据共享), care model (服务模式), dual matching (双向匹配), and health poverty alleviation (健康扶贫), etc.

#6 Two-way referral (双向转诊)	74	0.852	2013	2005–2013: Two-way referral (双向转诊), regional coordination (区域协调), and hierarchical diagnosis and treatment (分级诊疗); 2013–2020: Green channel (绿色通道), primary care (基层医疗), and health care information system (医疗卫生信息系统)

#7 Integration (整合)	69	0.859	2014	2009–2020: Integration mechanism (整合机制), medical insurance settlement (医保结算), equal emphasis on Chinese and Western medicine (中西医并重), healthy China strategy (健康中国战略), incentive plus constraint (激励约束), preliminary model exploration (模式初探), hierarchical diagnosis and treatment system (分级诊疗体系), family physician contracting system (家庭医生签约制度), contractual (契约式), medical–insurance–medicine Linkage (三医联动), rural integration path (农村整合路径), and people-oriented (以人为本), etc.

#8 Integrated health care (整合服务)	64	0.885	2016	2010–2016: Integrated health care system (整合型卫生服务体系), payment method of medical insurance (医疗保险的支付方式), health integration (健康整合), and healthy China (健康中国); 2016–2020: People-oriented (以人为本), patient-centered (以患者为中心), multi-level regional health care centers (多层级区域医疗卫生中心), and value-based care (价值医疗);

#9 Medical community (医共体)	63	0.84	2015	2006–2016: Chronic diseases (慢性疾病), contracted family physician service (家庭医生签约服务), operating mechanism (运行机制), and medical insurance payment (医保支付); 2016–2020: Incentive mechanism (激励机制), integrated management (一体化管理), informatisation (信息化), prevention first (预防为主), and resource sharing (资源共享)


*Note*: Size is the number of keyword nodes in the cluster. Silhouette value is a parameter used to evaluate the clustering effect; specifically, it evaluates clustering by measuring the network homogeneity index. Silhouette values closer to 1 indicate higher network homogeneity. Silhouette = 0.7 indicates that the clustering result has high reliability, and silhouette >0.5 indicates that the clustering result is logical.

Combining the results from Figure 1 and Table 1 indicates the following:

#0 Resource integration, #5 Medical resource integration: Although research continued from 2007 to 2020, there were no distinct keyword bursts, indicating that resource integration is less likely to become a future research trend.

#1 Longitudinal integration: Although the medical consortium bursts based on patient preferences were relatively small, the burst time was 2020; thus, this keyword will become the main hotspot and research trend in the longitudinal integration cluster.

#2 Integrated type, #7 Integration: Integrated research began to increase in 2005, with a few articles. Keywords such as integrated health care service and public hospital reform appeared to burst from 2007, followed by the integration transition period burst in 2009. Then, frequent keyword bursts emerged in 2016, including reform of the integrated health care system, the ‘Healthy China’ strategy, hierarchical diagnosis and treatment, county-level medical community and medical alliances. There were five keyword bursts in 2016–2020, indicating that research on integration type and integration continued to be popular and will become a major research trend, with many keywords. In terms of time span, integration has been studied in the health care system of China for approximately 20 years and is still receiving attention from researchers. Although this indicates that integration is vital for the health care system in China, it also suggests that there is considerable scope for research on integrated care in China, and further in-depth studies are needed to achieve breakthroughs.

#3 Combination of medical and elderly care: This cluster has been studied in China for a relatively short period; the earliest study appeared in approximately 2010. Informatisation of medical and elderly care in this cluster will be a continuing research trend.

#4 Medical consortium: This cluster had two large keyword bursts during 2007–2013, indicating that the number of articles on medical consortia increased sharply over this period. Bursts of effective approach, symbiosis theory and sharing management mechanism appeared in 2020, indicating that sharing and symbiosis theory may be an effective approach to address the challenges of constructing medical consortia in China.

#8 Integrated health care: Studies on integrated health care gradually increased from 2010, mainly exploring the integrated care models in Canada and the United States, which China learned from. In 2015, relatively more studies were reported on the mechanisms of integrated care in China, such as the complex adaptive system mechanism. Patient-centred, value-based care was mainly studied in 2020.

#### Keyword burst detection analysis

**Figure 2:**
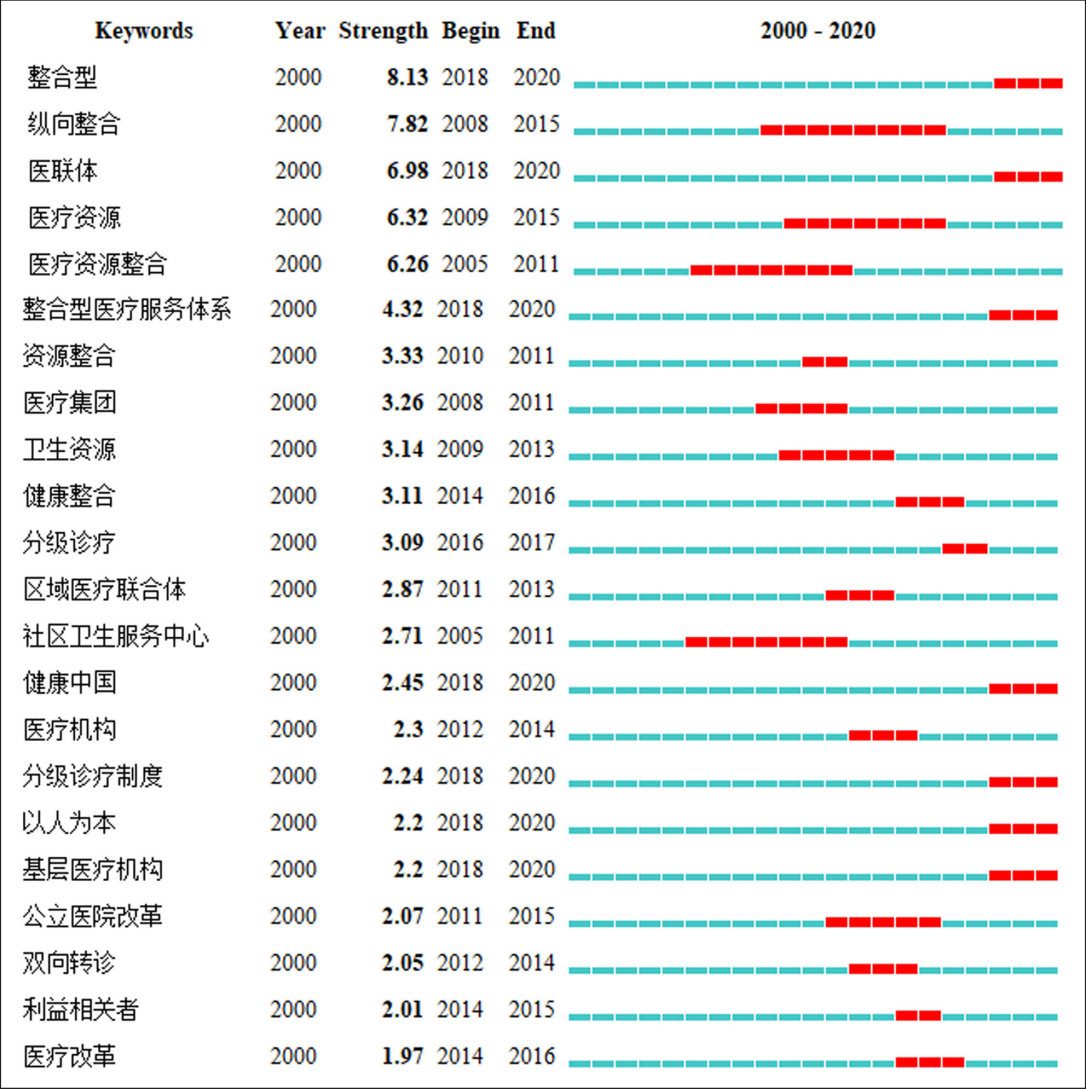
Burst detection of integrated care research in China.

The burstness function was used to detect keyword bursts. Figure 2 shows the emerging research trends in integrated care in China according to the time of keyword bursts detected and the burst strength rank.

Longitudinal integration had a burst strength of 7.82 (2008–2015), medical resources had a burst strength of 6.32 (2009–2015), medical group had a burst strength of 3.26 (2008–2011) and public hospital reform had a burst strength of 2.07 (2011–2015); these results indicate that associated research is becoming less attractive and research popularity has declined. In contrast, integrated type had a burst strength of 8.13 (2018–2020), medical consortium had a burst strength of 6.98 (2018–2020), integrated health care system had a burst strength of 4.32 (2018–2020), ‘Healthy China’ had a burst strength of 2.45 (2018–2020), hierarchical diagnosis and treatment system had a burst strength of 2.24 (2018–2020) and people-oriented care had a burst strength of 2.2 (2018–2020); accordingly, these research frontiers will continue to be research trends and researchers will pay increasing attention to these topics.

### Visual analytics of the English language literature

#### Co-citation network analysis

**Figure 3:**
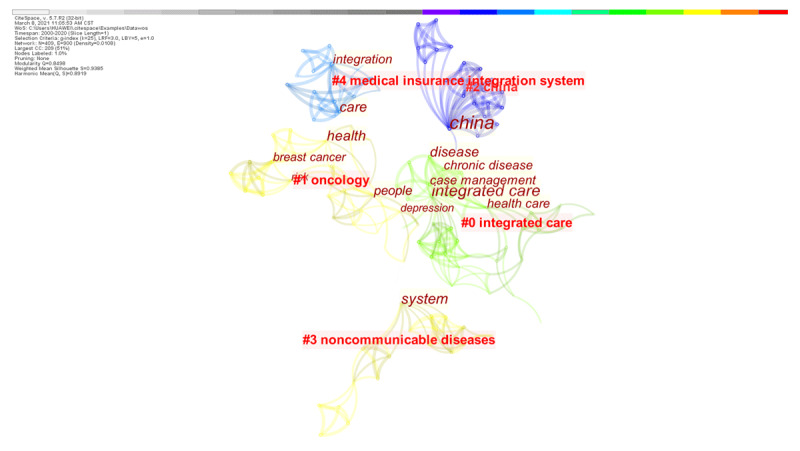
A visual map based on keyword cluster analysis of integrated care research in China.

The 124 articles exported from the Web of Science database were subjected to keyword cluster analysis (Figure 3), which generated five clusters. The network modularity Q = 0.8498 > 0.3, which indicates that the clustering was significant. The mean silhouette S = 0.9385 > 0.7, indicating that the cluster members were highly consistent and the results were convincing. In addition, the keyword nodes N = 409 and the connection number E = 900, with a network density of 0.0108. The largest CC indicates the information of 209 members in the largest subnetwork, which accounts for 51% of the total nodes. The most common keywords included ‘China’ (21 times), ‘integrated care’ (18 times), ‘system’ (8 times), ‘health care’ (4 times), ‘people’ (4 times) and ‘chronic disease’ (2 times).

Taken together, Figure 3 and Table 2 indicate that the English language literature on integrated care research in China mainly introduces the integrated care models and practices in China, the management of chronic diseases using integrated care models, medical insurance integration and payment method reforms. The popular topics and trends in the English language literature on integrated care research in China are similar to those in the Chinese language literature, but the content of the Chinese literature is richer than that of the English literature. Accordingly, Chinese researchers need to strengthen the academic promotion of research on integrated care in China.

**Table 2:** Summary of thematic concentrations in the English language literature.


CLUSTER	SIZE	SILHOUETTE	AVERAGE YEAR	KEYWORDS BURSTNESS	CITING ARTICLE (COVERAGE, %)

#0 integrated care	18	0.917	2015	integrated care model, medical consortium influence health outcomes, integrated prospective payment program, effectiveness, chronic diseases	Xin, W et al. 2018 [20] (9%) Sun, X. et al. 2014 [21] (8%)

#1 oncology	11	0.837	2017	expanded access, ovarian cancer patients, staff contextualise experiences, rural China, cross-sectional study, poverty	Li, Z.; Zhang, L. 2020 [22] (6%)

#3 noncommunicable diseases	11	0.985	2016	allocating ancillary service costs, cooperative game-based mechanism, uncontrolled chronic conditions, social health insurance consolidation	Cai, M. et al 2018 [23] (5%) Fei, H.; Hongyu, L. 2018 [24] (4%)

#4 medical insurance integration system	10	0.947	2011	catastrophic health expenditure incidence and its equity in China, a study on the initial implementation of the medical insurance integration system	Liu, C. et al 2018 [25] (4%) Liu, H. et al 2019 [26] (4%)


*Note*: Size is the number of keyword nodes in the cluster. Silhouette value is a parameter used to evaluate the clustering effect; specifically it evaluates clustering by measuring the network homogeneity index. Silhouette values closer to 1 indicate higher network homogeneity. Silhouette = 0.7 indicates that the clustering result has high reliability and silhouette >0.5 indicates that the clustering result is logical. The percentage of coverage = the percentage of references cited by a citing article.

#### Keyword burst detection analysis

**Figure 4:**
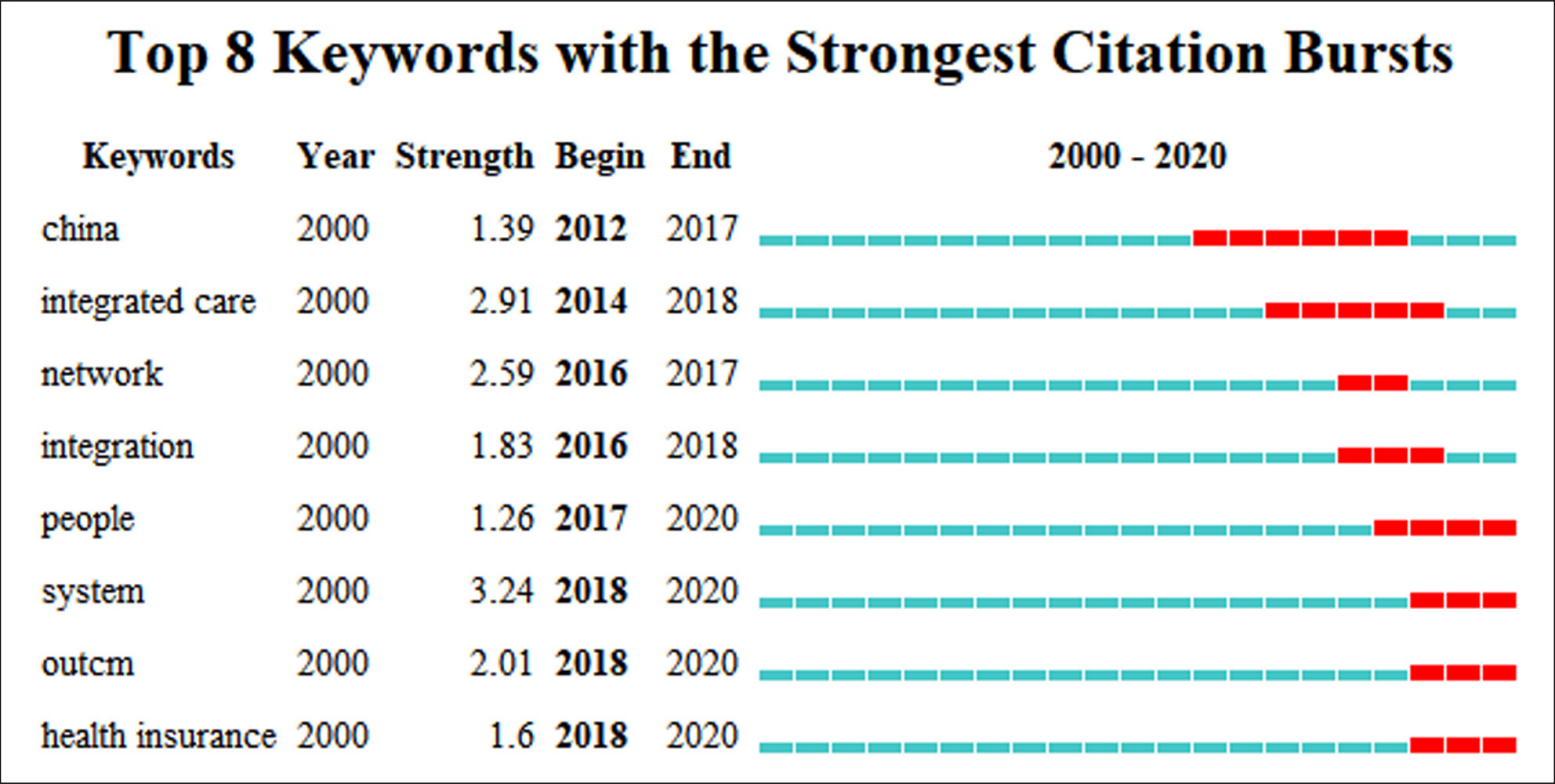
Keywords with the strongest frequency bursts in the core dataset.

Figure 4 shows the eight keywords that had the strongest bursts in publications during 2000–2020. Most of the keywords appeared to burst in recent years, mainly between 2016 and 2018. The strongest keywords included system, integrated care, network and outcome. Subsequently surged keywords included integration, health insurance, China and people. The most recent keyword bursts were people, system, outcome and health insurance. These results show that in publications in the English language database, researchers paid more attention to people-oriented system integration regarding integrated care research in China. Second, they continually estimated the cost-effectiveness of using integrated care and measured the integrated care health outcomes for Chinese patients. Third, they urged integrated care to be more synthesized with the health insurance payment method.

## Discussion some useful data

### Three stages of integrated care research in China over the last 20 years

The keyword clustering and timeline view analysis indicated that the research on integrated care in China has mainly comprised three stages. In the first stage (2000–2007), the main research keywords are resource integration, urban–rural two-way referral and longitudinal integration of medical resources [26,27]. This period is the start-up stage of integrated care research in China, and is characterized by very few keywords. In the second stage (2008–2015), the main research contents are continuous service, county-level medical community, integrated model, hierarchical diagnosis and treatment, medical consortia, medical alliances, coordinated service, benefits of integration of different levels, integrated perspective, paths to integration, transition period and incentive mechanisms [20,28,29]. This period is the emergence stage of integrated care research in China, with ongoing exploration of models and theories applicable to integrated care in China to coordinate the interests of multiple parties. In the third stage (2016–2020), the research keywords primarily include status quo analysis, influencing factors, practical dilemmas, breakthrough paths, fragmentation, effective approaches, patient preferences, shared management mechanisms, symbiosis theory, value-based care, payment methods and people-oriented care [19,30,31]. This period is the maturation stage of integrated care research in China. That is, there are still problems such as fragmentation and lack of synthesis in the implementation of integrated care in China. Based on existing studies of integrated care in China, researchers have proposed effective approaches, such as people-oriented care, value-based care and sharing/symbiosis, to address these challenges in the practice of integrated care.

### Leading role of academic research in policymaking in integrated care in China

Academic research on integrated care in China will help to shape lead policymaking for system integration at the macro level, institutional integration at the meso level and clinical integration at the micro level. In addition, integrated care research on value-based care will inform the policymaking process for patient management to achieve accessibility, quality management to achieve safety and cost management to achieve payment sustainability. Therefore, integrated care research in China clarifies and synthesises previous research experience, identifies popular and timely research topics and predicts future research trends. Further, such research directs policymaking on legal status, strategic planning, disciplinary frameworks, quality management, payment method reform-associated expenses and revenues and cost management in the integrated care system in China. This will ultimately develop an integrated care system that is high quality, efficient, care-integrated, safe, accessible and cost-controllable, and that will regenerate the health care delivery system in China.

### Prediction of future research trends in the field of integrated care and the possibility of policymaking

As the reform of the health care system in China progresses, we predict that the next stage of the research trend in integrated care in China will mainly focus on the theme of development goals and the ‘Healthy China’ strategy, patient preferences, shared management mechanisms, symbiosis theory, value-based care, payment methods and people-oriented care. Synthesising the keywords predicted by this study, we suggest that a regional integrated health care system with coordinated medical insurance and medical services could be constructed. First, a continuous, coordinated and lifecycle health service could be provided through integration, with the goal of promoting national health based on the principles of patient preferences and people-oriented care. Second, regional coordinated development and enhancement of primary care could be achieved by shared management mechanisms guided by symbiosis theory. Next, the organized collaboration and cost management of medical institutions could be guided by the reform of medical insurance payment methods. Finally, clinical, institutional and system integration could be achieved that incorporates integrated care and value-based care.

### Innovations and Limitations

The main innovations of this study are the identification of the evolution of integrated care research in China over the last 20 years and the prediction of future research trends based on scientometric analysis methods, such as keyword cluster analysis, timeline view analysis and burst detection analysis, using the CiteSpace software. The scientometric analysis showed that integrated care research in China is characterised by three stages: start-up, emergence and maturation.

There were some study limitations. First, although we searched two large databases (CNKI and Web of Science) to review Chinese and English language literature, some books and non-Chinese and non-English publications were not included. Because of the selection of search methods and exclusion criteria, some important research findings may have been missed. Therefore, our results should be treated with caution. Second, owing to the lack of reference data in the CNKI database, we did not perform a co-citation analysis, analyse specific references or perform a deeper analysis of the Chinese literature regarding important transitions. To ensure consistency between the analysis of the Chinese and English literature, we did not perform a co-citation analysis on the articles retrieved from the Web of Science database either. Therefore, additional research would be useful to further explore the English language literature to better understand the research progress on integrated care research in China in the international research environment. Third, the results of the CiteSpace software analysis were only checked by the researchers in our team; we did not consult experts from other research teams. Therefore, there may have been small omissions or variations in the study results. However, the overall findings and conclusions are consistent with the published paper about the lessons and outlook of integrating health and care in China [32], indicating the high credibility of our findings.

## Conclusions

Through an analysis of literature on integrated care in China, we clarified the main researchers and the evolution of keywords over the last two decades (2000–2020) and predicted future research trends. Integrated care is an effective approach to address fragmentation in health care systems. However, there are problems in the practice of integrated care in China. For example, medical institutions at different levels consider their own interests, so that patients do not really experience a coordinated, seamless and lifecycle provision of integrated care services. Hence, future studies on integrated care in China will need to likely focus on patient-centred, people-oriented value-based care, in addition to sharing and symbiosis. Integrated care theory may well gain knowledge will learn from academic concepts in the field of public management, such as value co-creation, to address the current problem of the lack of synthesis in integrated care in China. This will eventually help to build a regional integrated health care system with coordinated medical insurance and medical services.

## Reviewers

Dr Teresa Burdett, Principal Academic in Mental Health Nursing, Programme Lead for BSc Mental Health Nursing, Professional Lead for MSc Mental Health Nursing, Academic Advisor for G10 RNDA, Unit Lead for Foundations of Integrated Care and Person-Centred Services, Bournemouth University, UK.

Ye Fan W. Glavin, PhD, Clinical Faculty, Case Western Reserve University, School of Medicine, USA & Honorable and Visiting Professor, Chinese Academy of Medical Sciences, Peking Union Medical College, School of Health Policy and Management, Beijing, China.
